# The Role of Sorting Nexin 17 in Cardiac Development

**DOI:** 10.3389/fcvm.2021.748891

**Published:** 2021-12-20

**Authors:** Yufei Wu, Yaqun Zhou, Jian Huang, Ke Ma, Tianyou Yuan, Yong Jiang, Maoqing Ye, Jun Li

**Affiliations:** ^1^School of Medicine, Tongji University, Shanghai, China; ^2^Department of Cardiology, Shanghai General Hospital, Shanghai Jiao Tong University School of Medicine, Shanghai, China; ^3^Department of Cardiology, Shanghai East Hospital, Tongji University School of Medicine, Shanghai, China; ^4^Department of Endovascular Surgery, The First Affiliated Hospital of Zhengzhou University, Zhengzhou, China; ^5^Department of Echocardiography, Fuwai Hospital Chinese Academy of Medical Sciences, Shenzhen, Shenzhen, China; ^6^Shanghai Key Laboratory of Clinical Geriatric Medicine, Department of Cardiology, Huadong Hospital Affiliated to Fudan University, Shanghai, China

**Keywords:** congenital heart defects, double-outlet right ventricle, embryonic lethality, outflow tract, sorting nexin 17

## Abstract

Sorting nexin 17 (SNX17), a member of sorting nexin (SNX) family, acts as a modulator for endocytic recycling of membrane proteins. Results from our previous study demonstrated the embryonic lethality of homozygous defect of SNX17. In this study, we investigated the role of SNX17 in rat fetal development. Specifically, we analyzed patterns of *SNX17* messenger RNA (mRNA) expression in multiple rat tissues and found high expression in the cardiac outflow tract (OFT). This expression was gradually elevated during the cardiac OFT morphogenesis. Homozygous deletion of the *SNX17* gene in rats resulted in mid-gestational embryonic lethality, which was accompanied by congenital heart defects, including the double-outlet right ventricle and atrioventricular and ventricular septal defects, whereas heterozygotes exhibited normal fetal development. Moreover, we found normal migration distance and the number of cardiac neural crest cells during the OFT morphogenesis. Although cellular proliferation in the cardiac OFT endocardial cushion was not affected, cellular apoptosis was significantly suppressed. Transcriptomic profiles and quantitative real-time PCR data in the cardiac OFT showed that SNX17 deletion resulted in abnormal expression of genes associated with cardiac development. Overall, these findings suggest that SNX17 plays a crucial role in cardiac development.

## Introduction

The heart is the first functional organ formed during vertebrate embryogenesis (1). Cardiac development mainly consists of four stages, namely, the cardiac crescent, heart tube, cardiac looping, and formation of the four-chambered heart, and involves coordination of multi-cell or multi-signaling interactions (2). Abnormal heart development has been associated with development of congenital heart defects (CHDs), which account for a third of all the major congenital anomalies (3). Notably, the highest birth prevalence of CHDs, 9.3 per 1,000 live births, has been reported in Asia (4). Strikingly, malformations of the cardiac outflow tract (OFT) and the great arteries account for ~30% of all the CHDs (5).

Previous studies have demonstrated the pathological relevance of sorting nexins (SNXs) family, a particular class of phox homology domain-containing molecules from mammals to yeasts (6), in cancer, pathogen invasion, inflammation, and Alzheimer's disease (1, 7). The SNXs member, sorting nexin 17 (SNX17), reportedly binds to NPxY sequence-containing receptor proteins, via the FERM domain, to modulate membrane receptor protein endocytosis and signal transduction (8). Results from *in-vitro* studies have shown that SNX17 is localized in early endosomes (9), where it promotes endocytosis in members of the low density lipoprotein receptor (LDLR) family (10) and accelerates recycling of LDL receptor-related protein 1 (LRP1) (11), jagged canonical notch ligand 1 (Jag1a) (12), apolipoprotein E receptor 2 (ApoER2) (13), integrins (8, 14), and T cell receptor (TCR) (14). Results from our previous study revealed that SNX17 deletion leads to embryonic lethality (15), whereas findings from a high-throughput chemically mutagenized study suggested the requirement of SNX17 for heart development (16). To date, however, the morphological details and molecular mechanisms underlying its action remain unknown.

In this study, we applied the CRISPR/CAS9-mediated global approach to knockout the *SNX17* gene in rats. Results indicated that SNX17 defeciency led to mid-gestational embryonic death, a phenomenon that was accompanied by severe cardiac defects and was associated with suppressed cell apoptosis in the OFT endocardial cushion. Results from RNA-sequencing (RNA-seq) analysis revealed that SNX17 deletion caused abnormal expression of genes associated with cardiac development. Taken together, these findings indicate that SNX17 plays a critical role in heart development.

## Results

### Sorting Nexin 17 Expression Profiles in Cardiac Development

**Figure 1:**
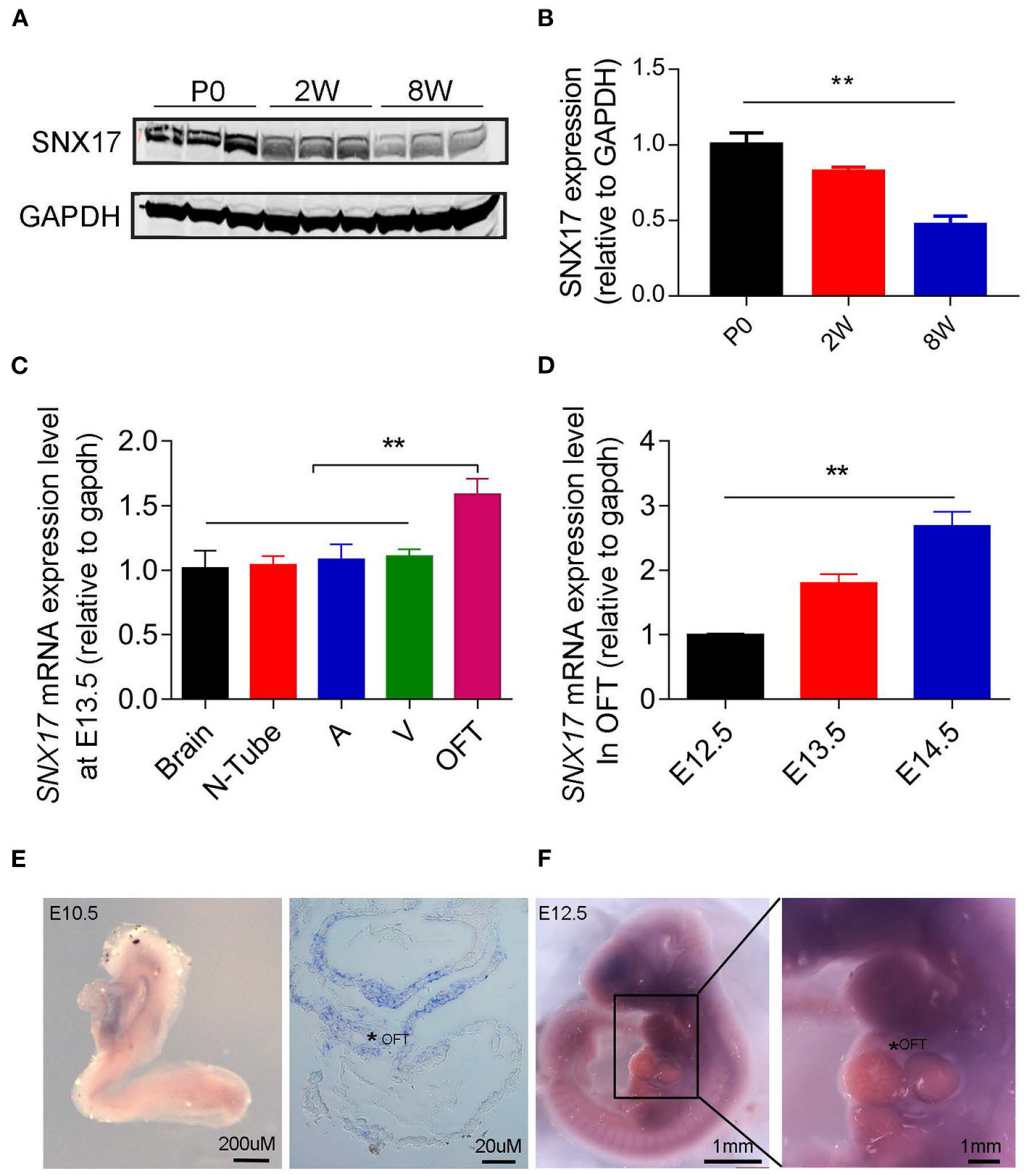
Profiles of *SNX17* expression in embryos **(A,B)**. Western blots showing levels of SNX17 protein in the heart of postnatal rats. *n* = 3 samples per group (*left*, representative blots; *right*, pooled data). **(C)** Expression of *SNX17* mRNA in multiple tissues at E13.5. *n* = 5 samples per group. **(D)** Expression of *SNX17* mRNA during the OFT morphogenesis (E12.5–E14.5). *n* = 5 samples per group. **(E)** Whole-mount *in-situ* hybridization for *SNX17* in a WT embryo at E10.5. Right panel, a representative microscopy image from a paraffin section (*, OFT). **(F)** Whole-mount *in-situ* hybridization for *SNX17* in a WT embryo at E12.5. *SNX17*, sorting nexin 17; mRNA, messenger RNA; WT, wild-type; N, neural tube; A, atrium; V, ventricle; OFT, outflow tract. **p < 0.01.

We analyzed expression profiles of SNX17 in rats to determine its potential role in the OFT development. Firstly, the expression level of SNX17 protein was gradually downregulated during postnatal heart development in rats (at day 0 and 2 and 8 weeks postnatal) (Figures 1A,B). Analysis of *SNX17* transcripts during embryonic development in the brain and neural tube (between the otic vesicle and the 9th somite) tissues and three heart regions from wild-type (WT) rat embryos at embryonic day 13.5 (E13.5) revealed that it was significantly upregulated in the cardiac OFT than in the brain, neural tube, atrium, and ventricles (Figure 1C). Next, we examined levels of *SNX17* messenger RNA (mRNA) expression of E12.5 to E14.5 rat embryos OFT. Interestingly, *SNX17* was upregulated during the cardiac OFT morphogenesis from E12.5 to E14.5 (Figure 1D), highlighting a close relationship between SNX17 and the cardiac OFT remodeling. To better demonstrate the expression pattern of *SNX17*, we used whole-mount *in-situ* hybridization (WMISH) technology, a classical method widely applied for analyzing gene expression across different cells, tissues, and organs. Strikingly, we detected specific *SNX17* expression in the cardiac OFT region at E10.5 (Figure 1E). However, *SNX17* was expressed in other regions during subsequent stages, although it was upregulated in the OFT region at E12.5 relative to the other cardiac regions (Figures 1C,F). Overall, these results suggest that SNX17 may be playing an important role in heart development.

### Ablation of Sorting Nexin 17 Leads to Embryonic Death Around Mid-Gestation

**Figure 2:**
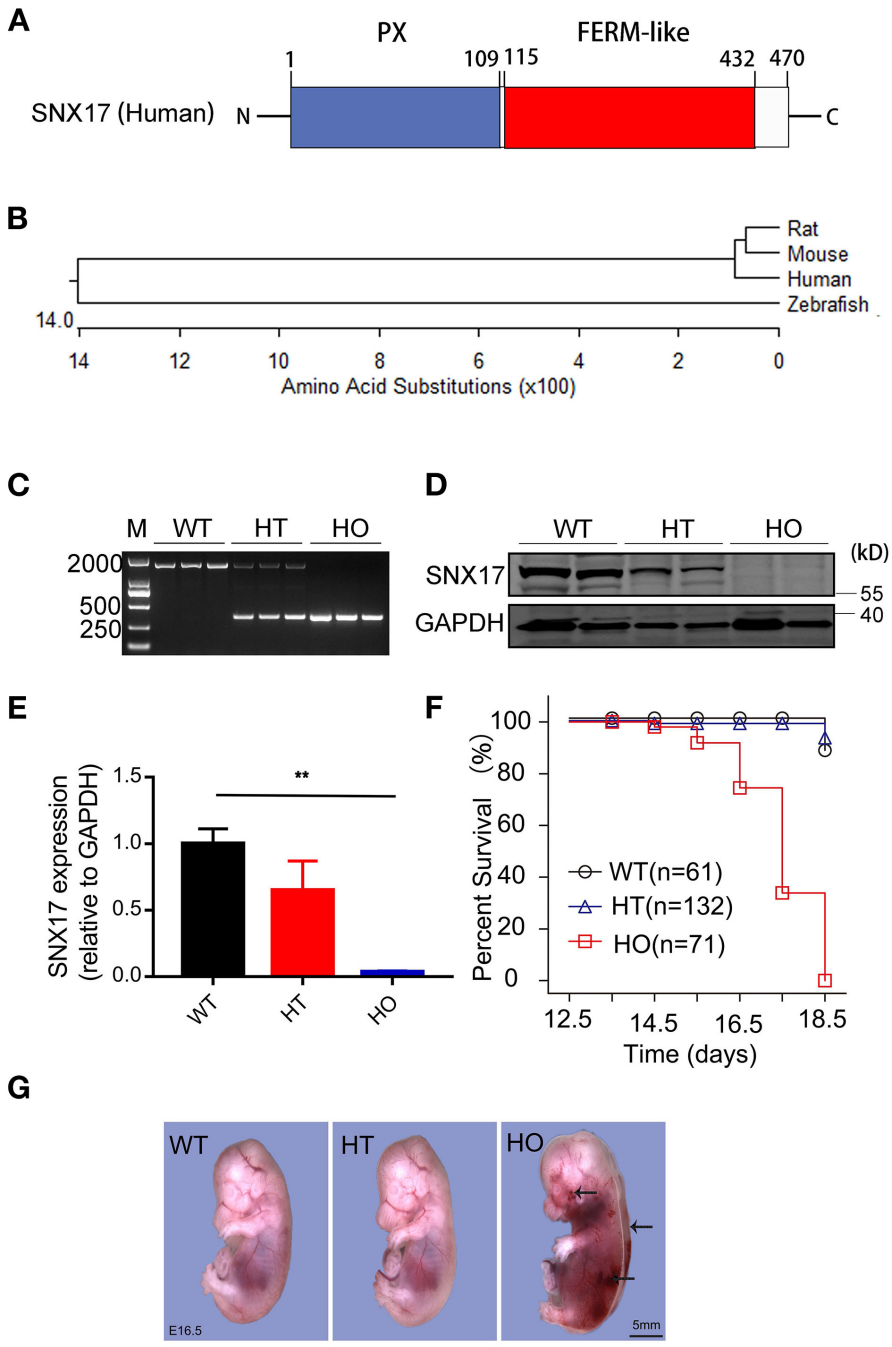
Ablation of SNX17 causes embryonic death around mid-gestation in rats. **(A)** A schematic representation of the human SNX17 structure. **(B)** Phylogenetic tree showing the relationship among different organisms. **(C)** Genotyping of SNX17 knockouts. **(D,E)** Western blots showing levels of the SNX17 protein at E16.5 (**D**, representative blots; **E**, pooled data). *n* = 3 samples per group. **(F)** Survival curve for embryos (E12.5–E18.5). A total of 264 embryos were genotyped. **(G)** Embryos at E16.5. Mutant embryo exhibited severe edema and hemorrhage (black arrow). M, DNA marker 2000; WT, wild-type; HT, heterozygous; HO, homozygous. **p < 0.01.

Human protein SNX17 consists of the classical phox (PX)- and FERM-like domains (Figure 2A). A SNX17 phylogenetic tree, generated via the MegAlign module of DNASTAR software (Clustal W method), revealed a high similarity between murines and human beings (Figure 2B). To determine the function of SNX17 *in vivo*, we applied CRISPR/CAS9 technnology to knockout the *SNX17* gene in rats (15) and obtained SNX17-null chimeras. Next, we crossed heterozygous (HT) F1 rats to generate homozygous (HO) offsprings, then genotyped them via PCR (Figure 2C). Thereafter, we validated efficiency of SNX17 deletion via protein expression analyses and found that SNX17 was significantly downregulated in HT and was almost absent in HO tissues (Figures 2D,E). Phenotypically, HT male and female rats appeared healthy with normal fertility and without significant overall growth retardation. However, HO embryos were embryonically lethal (Table 1). To further define the time window of embryonic death, we analyzed survival rates of HO embryos at different gestational stages and assigned them into three groups, namely, normal, ill (appearing edematous or hemorrhagic), and death, based on the appearance of HO embryos (Table 1). Results revealed that genotypic ratios of the survived embryos at stages of E12.5 and E13.5 were in line with the classic Mendelian ratio (Table 1) and all the HT and HO embryos were grossly normal. Notably, HO embryos began to die at E14.5 and no live HO embyos were observed at E18.5 (Figure 2F). In addition, almost half of all the HO embryos died at E17.5, thus the whole-embryo appearance was first analyzed at E16.5. Results revealed severe edema in all the freshly isolated HO embryos at E16.5, which was mainly around both the sides of the spinal cord. Moreover, some of them were hemorrhagic in the skin, especially near the neck (Figure 2G and Supplementary Figure 1). All the E16.5 viable embryos appeared edematous (19/19), while the incidence of focal and large-area hemorrhage on skin only accounted for 21.05% (4/19). At E15.5, there were also no embryos with hemorrhagic spots on the skin (*n* = 7).

**Table 1:** Genotype analysis of offspring from *SNX*17^+/−^ crosses.

**Age**	**+/+(*d*)**	**+/−(*d*)**	**−/−(*n-i-d*)**	**ND**
Postnatal	54	103	0	0
E18.5	8 (1)	18 (1)	4 (0-0-4)	0
E17.5	4	12	7 (0-1-6)	1
E16.5	18	39	26 (0-19-7)	2
E15.5	9	17	11 (1-7-3)	1
E14.5	5	7 (1)	4 (3-0-1)	2
E13.5	9	18	8 (8-0-0)	0
E12.5	9	21	11 (11-0-0)	1

*n-i-d, meaning the number of “normal, ill, dead” embryo; ND, genotype could not be determined due to technical difficulties*.

### Sorting Nexin 17-Null Rat Embryos Exhibit Prominent Cardiac Defects

**Figure 3:**
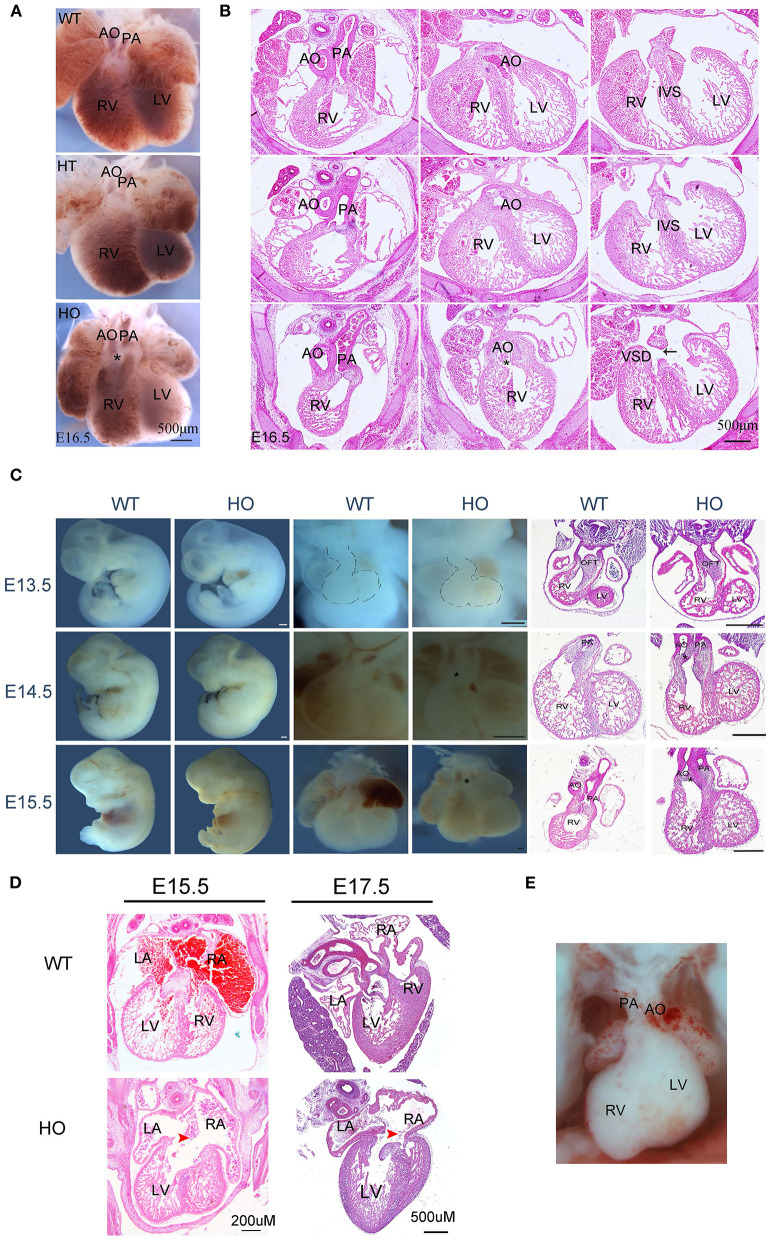
SNX17-null rats exhibit severe cardiac defects. **(A,B)** Representive images of the ventral heart and corresponding H&E sections from WT, HT, and HO embryos at E16.5 (black arrow, VSD). **(C)** Embryo morphology, heart, and H&E sections at E13.5 to E15.5 (*, DORV; all scale bar, 500 μM). **(D)** H&E staining images of AVSD defect from embryos at E15.5 and E17.5. **(E)** The DOLV defect image. AO, aorta; PA, pulmonary artery; RV, right ventricle; LV, left ventricle; LA, left atrium; RA, right atrium; IVS, interventricular septum; VSD, ventricular septal defect; DORV, double-outlet RV; DOLV, double-outlet LV.

Embryonic death is often attributable to cardiovascular system malformations (2). In this study, expression profiles of SNX17 protein indicated that it could be involved in heart development, especially the cardiac OFT. Consequently, we analyzed the cardiac structure. Microscopic images of the heart showed that at E16.5, aortic and pulmonary arteries in HO embryos were parallel and coursed vertically upward, compared to WT and HT ones, which resulted from different hemodynamics induced by the abnormal OFT alignment (Figure 3A). Subsequently, histological analysis showed that HO embryos exhibited double-outlet right ventricle (DORV) with ventricle septation defect ventricular septal defects (VSD), while the other embryos exhibited a normal cardiac structure (Figure 3B).

The division of the primitive single cardiac OFT into the aorta and pulmonary artery has previously been reported from E11.5 to E14.5 in mice (17), corresponding to E13.5–E16.5 in rats. Based on this, we further applied cross-sectional histological analysis to evaluate rat hearts from E13.5 to E15.5. Results of the heart morphology revealed a markedly abnormal spatial location of large arteries in HO embryos at E14.5 and E15.5 stages (Figure 3C). Histological analysis suggested that the OFT septation began at E13.5, with no obvious changes in the cardiac structure; however, the OFT anomaly appeared at later periods (Figure 3C). Additionally, we detected atrioventricular septal defects (AVSDs) in some HO embryos (Figure 3D). Next, we further analyzed penetrance for any abnormal phenotype, especially DORV and AVSD, after E13.5. Results revealed the presence of the DORV defect in 92.31% of HO embryos (12/13, *n* = 13), but only one of them exhibited double-outlet left ventricle (DOLV) (Figure 3E). However, no overriding aorta (tetralogy of Fallot) defects were detected in HO embryos. Moreover, penetrance of the AVSD defect was up to 66.67% in HO embryos (4/6, *n* = 6).

Previous studies have shown that the *SNX17* gene is expressed in the neural system, and its deficiency is found to affect neurogenesis in zebrafish (12). In addition to the heart, we also detected fetal brain structure between WT and HO embryos at E16.5 (Supplementary Figure 2). However, no obvious defects were observed in the head.

### Sorting Nexin 17 Knockout Does Not Affect Migration and the Number of Cardiac Neural Crest Cells, but Suppresses Mesenchymal Cell Apoptosis in the Cardiac OFT Tissue

**Figure 4:**
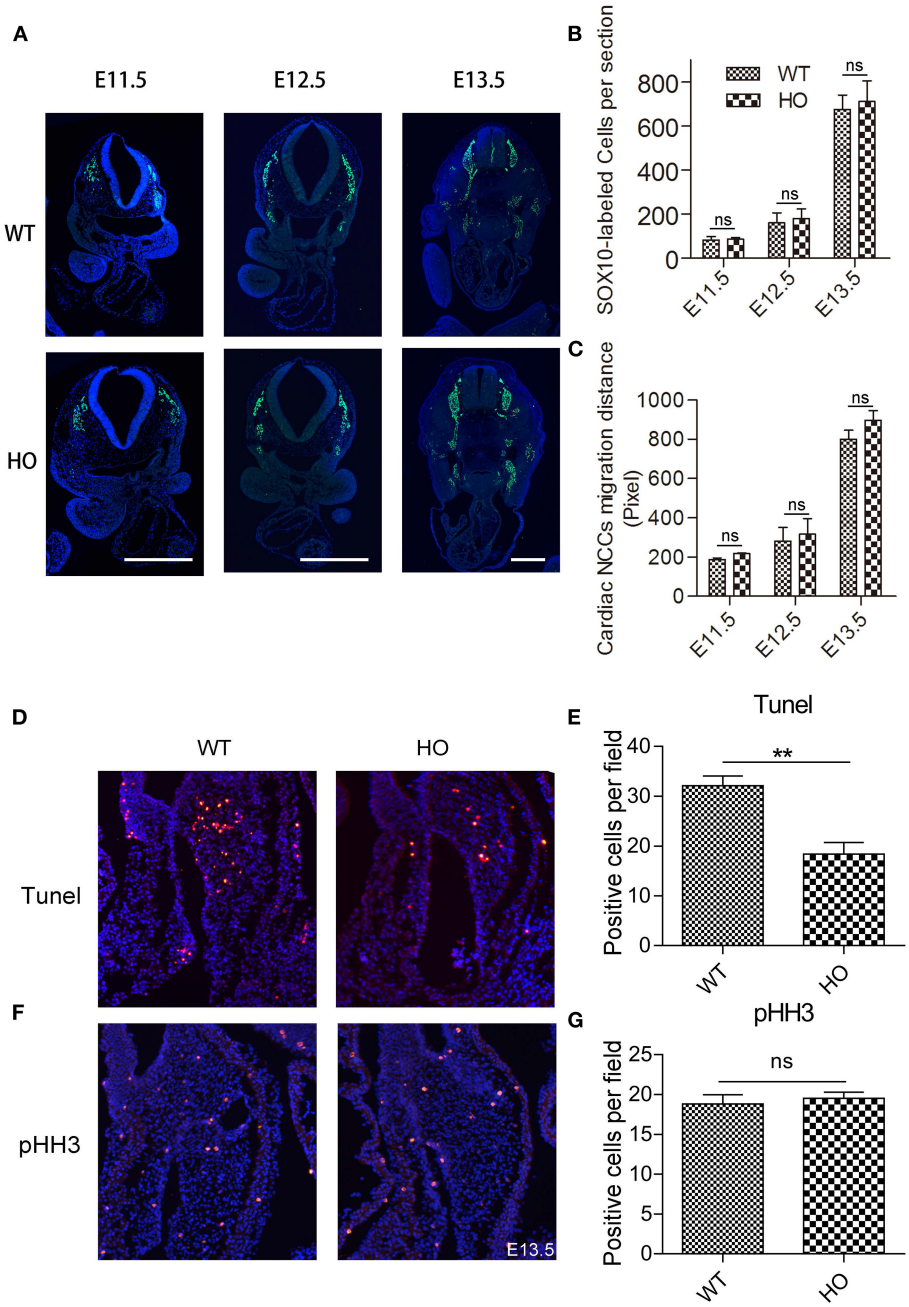
Knockout (KO) of SNX17 does not affect migration and the number of cardiac neural crest cells (cNCCs), but suppresses cellular apoptosis in the cardiac OFT cushion. **(A)** Sox10-labeled cNCCs in sections from embryos at E11.5 to E13.5. **(B,C)** Quantification of Sox10-labeled cNCCs number and migration distance. *n* = 6 embryos per group. **(D)** Results from terminal deoxynucleotidyl transferase (TdT)-mediated dUTP nick-end labeling (TUNEL) assay showing significant suppression of cell apoptosis in HO relative to WT. **(E)** Quantification of TUNEL-positive cells for data were shown in **(D)**. *n* = 6 embryos per group. **(F)** Cell proliferation measured by phosphohistone H3 (pHH3) immunohistochemical staining was significantly unchanged between HO and WT rats. **(G)** Quantification of pHH3-positive cells. *Blue*, DAPI. *n* = 6 embryos per group. **p < 0.01.

The characteristic migration of cardiac neural crest cells (cNCCs) plays an indispensable role in the OFT development (18). Reports have also shown that embryos with a neural crest deletion exhibit persistent truncus arteriosus, aortic arch interruption, DORV, or single ventricle and tricuspid valve anomalies (19). Generally, cNCCs migration begins from the dorsal neural tube at E8.5 in mice corresponding to E10.5 in rats. Subsequently, they migrate through the caudal pharyngeal arches into the OFT (17, 20). Therefore, we used anti-sex-determining region Y-box 10 (Sox10) immunofluorescence histochemistry to analyze the potential changes in migration distance and the number of cNCCs *in vivo* during the OFT development. Notably, there were no significant differences in either migration distance or cell number of cNCCs between WT and HO embryos from E11.5 to E13.5 (Figures 4A–C). Apart from cNCCs, resident local cells in the OFT region, derived from second heart field (SHF), have also been shown to play an essential role in the OFT remodeling (21). Our results indicated that *SNX17* was expressed in the SHF-derived cell cushion at E10.5 before cNCCs migrated to the OFT (Figure 1E). Previous studies have also implicated the imbalance between cellular apoptosis and proliferation in development of the cardiac OFT defects (22). To better understand the cellular basis of the OFT malformation observed in HO embryos, we evaluated cellular apoptosis and proliferation at an early stage of the ventricular OFT remodeling (E13.5) using terminal deoxynucleotidyl transferase (TdT)-mediated dUTP nick-end labeling (TUNEL) and anti-phosphohistone H3 (pHH3) immunohistochemistry, respectively. Results showed no significant change in proliferation between WT and HO embryos at this stage. However, cellular apoptosis was remarkably suppressed in HO embryos (Figures 4D–G).

### Transcriptomic Analysis Reveals the Underlying Mechanism of Cardiac Double-Outlet Right Ventricle Defect

**Figure 5:**
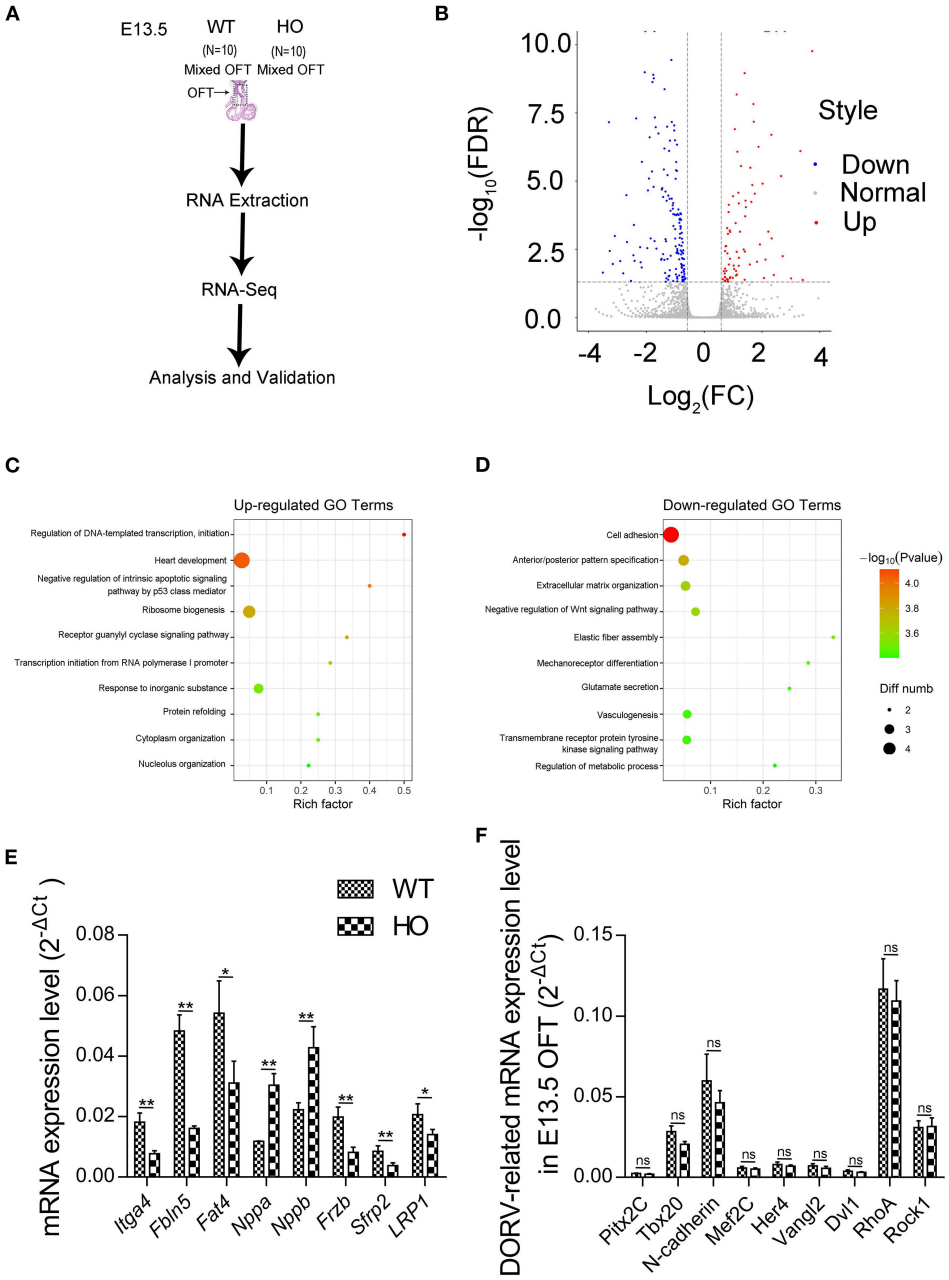
Transcriptomic analysis reveals the mechanism underlying the cardiac OFT defect in SNX17-KO embryos. **(A)** Experiment design. **(B)** Volcano plot illustrating differentially expressed genes (DEGs) of the cardiac OFT isolated from E13.5 mutants and control embryos. Red and blue dots denote upregulated and downregulated differentially expressed genes, respectively. **(C)** The top 10 most enriched GO terms related to biological processes for upregulated DEGs (sorted by *p*-value size). Rich factor refers to the GO term enrichment, a ratio of the number of the GO term A DEGs in our RNA-seq data to the number of all genes of the GO term A in the GO database. **(D)** Top 10 most enriched GO terms related to biological processes for downregulated DEGs. **(E)** Some genes involved in heart development, cell adhesion, and the Wnt signaling pathway were verified by qRT-PCR. Significant differences were observed in levels of mRNA expression between WT and HO OFTs. *n* = 5 per groups. **(F)** No significant differences were observed in levels of mRNA expression of DORV-related genes. *n* = 5 per groups. GO, Gene Ontology; qRT-PCR, quantitative real-time PCR. *p < 0.05; **p < 0.01.

We performed RNA-seq analysis to elucidate the potential mechanisms underlying development of the cardiac OFT defects induced by SNX17 knockout. Previous studies have shown that the OFT morphogenesis begins at E11.5 in mice, corresponding to E13.5 in rats (17). Accordingly, transcriptomic analysis was conducted at E13.5 in the cardiac OFT tissues isolated from WT and HO embryos (Figure 5A). A total of 262 differentially expressed genes (DEGs) were identified between WT and HO samples, of which 94 and 168 DEGs were upregulated and downregulated, respectively (Figure 5B). Analysis of biological functions of the DEGs, via the Gene Ontology (GO), revealed that the upregulated DEGs were mainly involved in heart development and negative regulation of the intrinsic apoptotic pathway, while the downregulated DEGs were associated with cell adhesion, extracellular organization, and negative regulation of the Wnt signaling pathway (Figures 5C,D and Table 2). Subsequently, we confirmed changes of some genes at the transcriptional level, especially those involved in heart development, cell adhesion, and the Wnt signaling pathway, using quantitative real-time PCR (qRT-PCR) (Figure 5E). In addition, we analyzed expression of some previously reported DORV-related genes, including *Pitx2c* (23), *Tbx20* (24), *N-cadherin* (25), *Mef2c* (22), *Her4* (26), *Vangl2* (27), *Dvl1* (28), *RhoA* (27), and *Rock1* (27) and found no statistically significant differences between WT and HO embryos (Figure 5F).

**Table 2:** Functional categorization of DEGs in biological process, relating to OFT remodeling.

**GO term/Gene name**	**Description**	**Log_2_(FC)**
*SNX17*	Sorting nexin 17	−0.51
**Cell adhesion**		
*Itga4*	Integrin, alpha 4	−0.93
*Fbln5*	Fibulin 5	−1.25
*Fat4*	FAT atypical cadherin 4	−0.75
*Siglec1*	Sialic acid binding Ig-like lectin 1, sialoadhesin	−2.91
*Lgals3bp*	Lectin, galactoside-binding, soluble, 3 binding protein	−1.09
*Pdzd2*	PDZ domain containing 2	−0.93
*Abl1*	ABL proto-oncogene 1, non-receptor tyrosine kinase	−0.77
*LOC100911597*	Myosin-9-like	−0.72
*Tmem8b*	Transmembrane protein 8B	−0.72
*Spon1*	Spondin 1, extracellular matrix protein	−1.04
*Nuak1*	NUAK family, SNF1-like kinase, 1	−0.83
*Susd5*	Sushi domain containing 5	−3.51
*Col6a6*	Collagen, type VI, alpha 6	−1.25
*Mfap4*	Microfibrillar-associated protein 4	−1.72
**Heart development**		
*Nppa*	Natriuretic peptide A	1.54
*Bmp10*	Bone morphogenetic protein 10	2.12
*Cacybp*	Calcyclin binding protein	0.71
*Bmp2*	Bone morphogenetic protein 2	0.79
*Nppb*	Natriuretic peptide B	1.15
*Cox17*	COX17 cytochrome c oxidase copper chaperone	0.81
**Negative regulation of intrinsic apoptotic signaling pathway by p53 class mediator**		
*LOC102554312/* *LOC100910121*	RNA polymerase I-specific transcription initiation factor RRN3-like	5.02 /1.24
**Negative regulation of Wnt signaling pathway**		
*Apc2*	Adenomatosis polyposis coli 2	−1.34
*Frzb*	Frizzled–related protein	−0.94
*Sfrp2*	Secreted frizzled-related protein 2	−0.97
*Lrp1*	Low density lipoprotein receptor-related protein 1	−0.69
**Extracellular matrix organization**		
*Fbln5*	Fibulin 5	−1.25
*Pdgfra*	Platelet derived growth factor receptor, alpha polypeptide	−0.70
*Eln*	Elastin	−0.94
*Crispld2*	Cysteine-rich secretory protein LCCL domain containing 2	−0.84
*Smoc2*	SPARC related modular calcium binding 2	−0.89
**Cardiocyte differentiation**		
*B9d1*	B9 protein domain 1	2.75
*Bmp2*	Bone morphogenetic protein 2	0.79
**Negative regulation of systemic arterial blood pressure**		
*Nppa*	Natriuretic peptide A	1.54
*Nppb*	Natriuretic peptide B	1.15
**Regulation of body fluid levels**		
*Nppa*	Natriuretic peptide A	1.54
*Pdss1*	Prenyl (decaprenyl) diphosphate synthase, subunit 1	1.10

## Discussion

This study expounded the biological function of SNX17, a member of FERM domain-containing SNX family, in mammalian fetal development. Cardiac defects in SNX17-null rats underscored the importance of SNX17 in cardiac development and were likely associated with embryonic lethality. Notably, results from deep sequencing of the transcriptome and experimental validation in the OFT tissues revealed the potential mechanism for the cardiac OFT morphogenesis modulated by SNX17.

Results from analysis using the *Lasergene 7* software and the national center for biotechnology information (NCBI) database revealed that deletion of sequences in the PX domain of the *SNX17* gene resulted in a frameshift mutation and loss of SNX17 function. Remarkably, we found that ablation of *SNX17* in rats resulted in heart defects, which was in contrast to the findings from a previous study which showed that morpholino knockdown of SNX17 in zebrafish led to developmental defects in neurogenesis and the pancreas (12). This discrepancy may be attributed to differences between the species analyzed and alignment of amino acid sequences from different organisms revealed that rats had a low SNX17 similarity with zebrafish.

The fetal development for mice includes four stages, namely, pre- and peri-implantation (E0–E4.5), differentiation of the germ layers (E6–E9), development of the cardiovascular system and placenta (E9–E12), and tissue and organ differentiation (E12–E19) (29, 30). The lethality induced by SNX17 deletion in rats happened mainly after E15.5 corresponding to E13.5 in mice. After E12 in mice, common causes of embryonic death include hematopoietic defects and heart defects (29). Large-area hemorrhage is often indicative of hematopoietic defects. However, the incidence of hemorrhage in HO embryos is not higher than heart defects. Moreover, our evidences suggested that the heart of HO embryos at E14.5 had been abnormal before the appearance of skin edema and hemorrhage. So, heart defects of HO embryos are independent of edema and hemorrhage. In addition, developmental defects initiated in any stage of gestation for brain, kidney, liver, and lung that may allow the embryos to survive before birth (29). At E12.5, the development of the placenta has completed. If the placenta fails to develop normally, embryonic growth would be restricted. However, the growth retardation in HO embryos was not found. Thus, as far as current data is concerned, heart defects are likely the most potential cause for embryonic death induced by SNX17 deletion.

Double-outlet right ventricle, a high-penetrance defect, was in almost all the SNX17-ablated embryos, which was reminiscent of the Taussig–Bing malformation, a DORV with a bilateral conus. During cardiogenesis, the primitive single cardiac OFT transiently connects the aortic sac to the right ventricle. Consequently, the cardiac OFT divides and then rotates to constitute the ascending aorta and pulmonary trunk base, a process termed as the OFT remodeling (31). During this process, the OFT septum is responsible for the OFT separation, while the aortic switch procedure is assocated with the OFT alignment (32). At the cardiac looping (D-loop) stage, the developing ascending aorta is placed to the right of the developing main pulmonary artery of the truncus arteriosus. Subsequently, an embryonic aortic switch procedure is performed, based on growth of the left-sided subpulmonary conal free wall and resorption of the right-sided subaortic conal free wall. Continued persistence and growth of both the subaortic and subpulmonary conal free walls, i.e., failure of subsemilunar conal free wall resorption, results in development of DORV. Moreover, incorrect arterial switch procedures cause other OFT malalignment, including transposition of the great arteries (TGA), tetralogy of Fallot (TOF), and DOLV, among others.

Previous studies have shown that migration and differentiation of cNCCs are required for the OFT morphogenesis (33). However, whether cNCC derivatives are responsible for cardiac defects induced by SNX17 defeciency remains unknown. In this study, we visually tracked Sox10-labeled cNNCs using immunofluorescence histochemistry from E11.5 to 13.5. However, we found no statistically significant changes in migration distance and the number of cNNCs in SNX17-null rats. Apart from cNCCs, the second heart field (SHF) has also been implicated in the OFT alignment (34). In this study, results from WMISH revealed that *SNX17* was expressed in SHF derivatives before the migration of cNCCs into the OFT, suggesting that SNX17 likely plays a key role in resident cell. Previous studies have demonstrated the importance of the balance between cell apoptosis and proliferation in cardiovascular development and morphogenesis (35) linked to the OFT malformation. For example, knockout of the *Rxra* gene in mice results in significant upregulation of apoptotic signaling in the endocardial cushion of the OFT, while the OFT and aortic arch appear malformed (36). Therefore, we investigated cell proliferation and apoptosis in the OFT in SNX17-null embryos and found that cell apoptosis potentially affected the OFT malalignment caused by SNX17 deletion rather than cell proliferation.

To elucidate the potential mechanism underlying the OFT malalignment, we performed RNA-seq analysis and found that SNX17 deletion led to dysfunction of some biological processes in the OFT, including heart development, cell adhesion, negative reulation of the Wnt, and apoptotic signaling pathways. Firstly, these results revealed significant differential expression of some genes that are correlated with biological processes, such as “heart development,” especially Nappa and Nappb, in line with previous studies that have reported that upregulation of Nppa and Nppb is associated with abnormal heart function (37). Secondly, cell adhesion was the most significantly downregulated blood pressure (BP) term. Previous studies have shown that SNX17 depletion results in inhibition of cell adhesion *in vitro* (8). However, data from our *in vitro* cNNCs experiments did not support this result. In addition to cNNCs, whether SNX17 deletion *in vivo*, affects local cells in the OFT, remains unclear. Therefore, further explorations are needed to unravel the exact role played by cell adhesion in the OFT remodeling investigated. Additionally, the evolutionarily conserved Wnt signaling pathway, which comprises three major comonents, namely, canonical, planar cell polarity, and Wnt/Ca^2+^, plays a crucial in regulation of heart development (38). Previous studies have shown that tissue-specific deletion of β-catenin (the core molecule in the canonical Wnt pathway) in Isl1- or Wnt1-expressing cells results in development of cardiac defects involving morphogenesis of outflow tract and formation of atrioventricular cushion and atrial septum (39, 40). Moreover, occurrence of mutations in the *Vangl2* gene, a key member of the non-canonical Wnt signaling pathways (planar cell polarity), has been implicated in DORV development (41), generating a similar phenotype observed in SNX17-null embryos. In addition, loss-of-function in transcription factor Pix2c results in DORV development and atrial septal defect (ASD) (42, 43). Results from our RNA-seq and qRT-PCR analyses revealed significant differences of some genes associated with the Wnt signaling pathway, namely, *Lrp1, Frzb, Sfrp2*, and *Apc2*. Notably, occurrence of missense mutations in the *Lrp1* gene has been found to cause DORV with AVSD (44), which was consistent with the SNX17-null phenotype, probably by interfering with cushion fibroblast motility and migration. Finally, abnormal regulation of the apoptotic signaling pathway, evidenced by RNA-seq data, may be associated with suppression of cell apoptosis in the OFT cushion. Collectively, SNX17 deletion can cause abonormal gene expression profiles in the cardiac OFT.

In summary, our findings demonstrate that SNX17 plays an indispensable role in cardiac development.

## Materials and Methods

### Rats and Embryos

Sorting nexin 17-null rats were generated using the CRISPR/CAS9 system as previously reported (15), then their chimeras crossed with WT Sprague–Dawley (SD) rats to produce F1 heterozygotes. Offsprings from F1 HT crosses were genotyped via PCR and validated using Western blotting. Detection of *SNX*17^−/−^ rats was done using the following primers: forward—5′-TTTTGACTGGGTCTTCTGT-3′ and reverse—5′-GATCAGTTTCCACGCTAGAT-3′, while reverse—5′-AGCCGTGATTAAAGGGTA-3′ was used to detect the WT allele. The day when a copulation plug was observed was designated as embryo 0.5 day (E0.5). Pregnant rats were anesthetized using intraperitoneal (IP) injection of pentobarbital (100 mg/kg) and their embryos collected *via* cesarean section. All the animal protocols were performed in accordance with the Guidelines for the Care and Use of Laboratory Animals and approved by the Institutional Animal Care and Use Committee at the School of Medicine, Tongji University.

### Western Blotting

Total proteins were extracted from cells or tissues using the radioimmunoprecipitation assay (RIPA) lysis buffer (P0013C, Beyotime, China) containing protease inhibitor cocktail (04693132001, Roche, Mannheim, Germany). Equal protein concentrations were separated on NuPAGE gels (Invitrogen, Carlsbad, CA, USA) and then transferred to polyvinylidene fluoride (PVDF) membranes (Millipore, Billerica, MA, USA). Membranes were blocked with 5% non-fat milk in tris-buffered saline solution with 0.1% Tween-20 (TBS-T) solution at room temperature for 2 h and then incubated overnight with anti-SNX17 (10275-1-AP, Proteintech, Rosemont, IL, USA) and glyceraldehyde-3-phosphate dehydrogenase (GAPDH) (60004-1-lg, Proteintech, Rosemont, IL, USA) antibodies at 4°C. The membranes were subsequently incubated with corresponding secondary antibodies, namely, donkey anti-rabbit immunoglobulin G (IgG) (H + L) (ab186693, Abcam, Cambridge, UK) and donkey anti-mouse IgG (H + L) (ab186699, Abcam, Cambridge, UK) for about 2 h at room temperature and then subjected to detection on the Odyssey IR Imaging System (Li-Cor, Lincoln, Nebraska, USA). Intensity of immunoblots was analyzed using Quantity One (Bio-Rad, Hercules, CA, USA), while relative protein levels were quantified using (ImageJ software).

### Quantitative Real-Time PCR

Cardiac OFT tissues from 10 embryos were collected and mixed prior to RNA isolation, whereas extraction on the other samples was performed on three individual embryos. Total RNA was extracted from the aforementioned tissues using the Trizol reagent (15596-026C, Invitrogen, Carlsbad, CA, USA), chloroform extraction, isopropanol precipitation, and precipitate washing in 75% ethanol and then quantified using a Nanodrop spectrophotometer (Thermo Fisher Scientific, Waltham, MA, USA). Complementary DNA (cDNA) was synthesized from the RNA using 5X PrimeScript RT-PCR Master Mix Kit (RR036A, Takara, Japan) following the instructions of the manufacturer, while the remaining RNA samples were stored at −80°C to await transcriptome analysis. qRT-PCR was performed using the SYBR^®^ Green Real-time PCR Master Mix (QPK-201, Toyobo, Japan), targeting specific genes given in Table 3. Rat *GAPDH* was used as an internal reference gene. Except for Figures 5E,F, relative expression levels of target mRNAs were calculated using the 2^−ΔΔCt^ method. The 2^−ΔC^ method was used to calculate the relative fold gene expression between different genes in Figures 5E,F. “ΔCt” means “CT _(Targetedgenes)_-CT _(GAPDH)_.”

**Table 3:** List of primers used in qPCR.

**Primer name**	**F/R**	**Sequence 5^**′**^-3^**′**^**	**Size (bp)**
*SNX17*	F	GAGTCCTGCACTGTCGTGTG	82
	R	AGCACATTGGCCCCATACTC	
*Itga4*	F	ACTTCGCAAGGTTTTGTGCC	196
	R	CCTGTGGGGAGTTGGACATT	
*Fbln5*	F	TGATGACACGCCCCATCAAA	141
	R	CCGAGGCTCAGAACGGATAC	
*Fat4*	F	GAGAGCCGGGTCATTCGTAG	110
	R	CATGCAGCTCGAAATCTGGC	
*Nppa*	F	CAACACAGATCTGATGGATTTCA	71
	R	CCTCATCTTCTACCGGCATC	
*Nppb*	F	GTCTCAAGACAGCGCCTTCC	132
	R	AACCTCAGCCCGTCACAGC	
*Sfrp2*	F	GATCACCTCCGTGAAACGGT	158
	R	CGGAAATGAGGTCGCAGAGT	
*Frzb*	F	CCCTTGAGCCACTGACAACA	196
	R	CTGGTCAATACAGCGAGCCA	
*Lrp1*	F	CTATCGGTCATCGGCAGCAT	177
	R	GATCAAGGGGCTGTGTCCAA	
*Pitx2C*	F	TCGCAGAGAAAGATAAGGGCCA	176
	R	GATTTCTTCGCGAGTGGACA	
*Tbx20*	F	GCTATGCCTATCACCGGTCC	173
	R	ATGGCCATGTTGGTCCAGTT	
*N-cadherin*	F	CACCCGGCTTAAGGGTGATT	185
	R	CGATCCTGTCTACGTCGGTG	
*Mef2C*	F	TGCTGGTCTCACCTGGTAAC	148
	R	ATCCTTTGATTCACTGATGGCAT	
*Her4 (Erbb4)*	F	GTACGAGCCTGCCCTAGTTC	104
	R	GTGCCGATTCCATCACATGC	
*Vangl2*	F	ACCAATGGGCTTAAGGACGG	162
	R	TCACACAGAGGTCTCCGACT	
*Dvl1*	F	CCAGAAAGGACCAGTGGCAT	132
	R	CCTAGGTCGGTCTCGTCTCA	
*RhoA*	F	AGGATTGGCGCTTTTGGGTA	136
	R	TTCACAAGATGAGGCACCCC	
*Rock1*	F	GCGGATCTTGCTACAGAGTGA	181
	R	TAACTCCCGCATTTGCCCTT	
*Gapdh*	F	CTGGTGCTGAGTATGTCGTGGA	198
	R	AGTTGGTGGTGCAGGATGCATT	

*F, Forward; R, Reverse*.

### Histological Analysis

Freshly-dissected embryo bodies were observed under a stereomicroscope. Parts of the embryos and embryonic hearts were fixed overnight with 4% paraformaldehyde (PFA) at 4°C and then embedded in paraffin wax or optimal cutting temperature compound (OCT). The paraffin sections were stained with H&E for detection of the cardiac structure. To analyze cell proliferation and detect apoptosis in the cardiac OFT, sections from HO or WT individual embryos at E13.5 were stained with anti-PHH3 (ab32107, Abcam, Cambridge, UK) (45) and *in situ* Cell Death Detection Kit, Fluorescein (11684795910, Roche, Mannheim, Germany). For detection of the cNNCs, the OFT-containing sections were incubated with anti-Sox10 primary antibody (ab227680, Abcam, Cambridge, UK), washed with phosphate buffered saline solution with 0.1% Tween 20 (PBST), and then incubated with a secondary antibody [goat antirabbit IgG (H + L) (A27039, Invitrogen, Carlsbad, CA, USA)] in fluorescence immunohistochemical assay. Finally, slides were mounted using DAPI medium (SL1841, Coolaber, Beijing, China) and then three discontinuous sections per embryo subjected to fluorescence microscopy on a Leica microscope (DM6000B, Leica, Germany). Signals were quantified using (ImageJ software), with 10 fields that were randomly selected from three cardiac sections used to quantify positive cell number from the fluorogram.

### *In-situ* Hybridization

Whole-mount *in-situ* hybridization was performed, as previously described (46). A digoxigenin-labeled antisense probe sequence (5'-CCCACCCTCCGACGCAGCATCTTCCTGATA-3') at both the ends of the probe was modified by digoxigenin. Next, embryos were fixed, dehydrated, rehydrated, and pretreated to increase accessibility of cellular RNA in the probe. Thereafter, embryos were hybridized with the riboprobe and then hybridization detected by anti-digoxigenin antibody coupled to alkaline phosphatase (ab119345, Abcam, Cambridge, UK). Finally, a signal was generated by incubating the embryos with NBT (11383213001, Roche, Mannheim, Germany) and BCIP (11383221001, Roche, Mannheim, Germany).

### Ribonucleic Acid Sequencing and Transcriptomic Analysis

Total RNA was extracted from the cardiac OFT samples of *SNX17*-knockout and WT embryos using Trizol reagent (Invitrogen, Carlsbad, CA, USA) separately. RNA quality was checked on the Agilent 2200 System (Agilent Technologies, Santa Clara, California, USA) and then RNA with a RNA integrity number (RIN) > 7.8 used to construct cDNA libraries using the Ion Total RNA-Seq Kit version 2.0 (4475936, Life Technologies, Carlsbad, CA, USA) according to the instructions of the manufacturer. The libraries were subjected to single-end sequencing (Novel Biomedicine Technology Corporation Ltd., Shanghai, China) and read cleaned then mapped onto the reference genome. The cDNA libraries were then processed for the proton sequencing process according to the commercially available protocols. Samples were diluted and mixed, the mixture was processed on a OneTouch 2 instrument (Life Technologies, Carlsbad, CA, USA), and enriched on a OneTouch 2 ES station (Life Technologies, Carlsbad, CA, USA) for preparing the template-positive Ion PI^TM^ Ion Sphere^TM^ Particles (Life Technologies, Carlsbad, CA, USA) according to Ion PI^TM^ Template OT2 200 Kit version 2.0 (Life Technologies, Carlsbad, CA, USA). After enrichment, the mixed template-positive Ion PI^TM^ Ion Sphere^TM^ Particles of samples was loaded onto 1 P1 verion 2 Proton Chip (Life Technologies, Carlsbad, CA, USA) and sequenced on proton sequencers according to Ion PI Sequencing 200 Kit version 2.0 (Life Technologies, Carlsbad, CA, USA). A total of 12.9 and 12.8 million raw reads were obtained from two libraries. The raw reads were cleaned by removing adaptor sequences, reads with > 5% ambiguous bases (noted as N) and low-quality reads, and the clean reads aligned to the rat genome (Rnor 6.0) using the MapSplice program (version 2.1.8). We applied the EBSeq algorithm to identify DEGs based on a false discovery rate (FDR) and the following criteria: (i) FC > 1.5 or < 0.67 and (ii) FDR < 0.05. The GO analysis was performed to elucidate the function of *SNX17* and understand the cardiac OFT defect.

### Statistical Analysis

Statistical analyses were performed using the SPSS software, IBM, USA, version 20.0 and all the data were expressed as mean ± SEM. Comparisons between two and multiple groups were analyzed using the unpaired Student's *t*-test and the one-way ANOVA with least significant difference (LSD) *t*-test, respectively. Statistical significances were considered at ^*^p < 0.05; ^**^p < 0.01; and ns, non-significant.

## Data Availability Statement

The data presented in the study are deposited in NCBI's Gene Expression Omnibus (GEO) database repository, accession number GSE186706. This data has been released, please go to https://www.ncbi.nlm.nih.gov/geo/query/acc.cgi?acc=GSE186706.

## Ethics Statement

The animal study was reviewed and approved by the Animal Care and Use Committee at the School of Medicine, Tongji University.

## Author Contributions

YW, JL, and MY designed the experiments and wrote the manuscript. YW, YZ, JH, KM, and TY carried out this experiment. YW analyzed the data. JL, MY, and YJ supervised this project. All authors contributed to the article and approved the submitted version.

## Funding

This work was supported by funds from the National Natural Science Foundation of China (Grant Nos: 81870293, 82070414, 91949126, and 81970378).

## Conflict of Interest

The authors declare that the research was conducted in the absence of any commercial or financial relationships that could be construed as a potential conflict of interest.

## Publisher's Note

All claims expressed in this article are solely those of the authors and do not necessarily represent those of their affiliated organizations, or those of the publisher, the editors and the reviewers. Any product that may be evaluated in this article, or claim that may be made by its manufacturer, is not guaranteed or endorsed by the publisher.
