# Supplementary material for: The Role of Sorting Nexin 17 in Cardiac Development
Source: Front Cardiovasc Med. 2021 Dec 20;8:748891. doi: 10.3389/fcvm.2021.748891 (PMC8720881; doi:10.3389/fcvm.2021.748891)
Supplement: Supplementary file 1 [file Data_Sheet_1.zip › Captions of Supplementary Figures.docx]

**Supplementary Figure 1** HO embryos at E16.5.

**Supplementary Figure 2** Head histological analysis of WT and HO embryos at E15.5. Anatomical structures are identified as follows: tv, third ventricle; fv, fourth ventricle; lv, lateral ventricle; cp, choroid plexus; pe, primitive ectomeninx; s, skin; nt, dorsal neural tube; fg, facial ganglion; tg, trigeminal ganglion; cc, cochlear canal; e, eye; jls, jugular lymph sac; pnc, primitive natal cavity; t, tongue. *, skin swelling.
