# Supplementary figures and images for: The Role of Sorting Nexin 17 in Cardiac Development
Source: Front Cardiovasc Med. 2021 Dec 20;8:748891. doi: 10.3389/fcvm.2021.748891 (PMC8720881; doi:10.3389/fcvm.2021.748891)

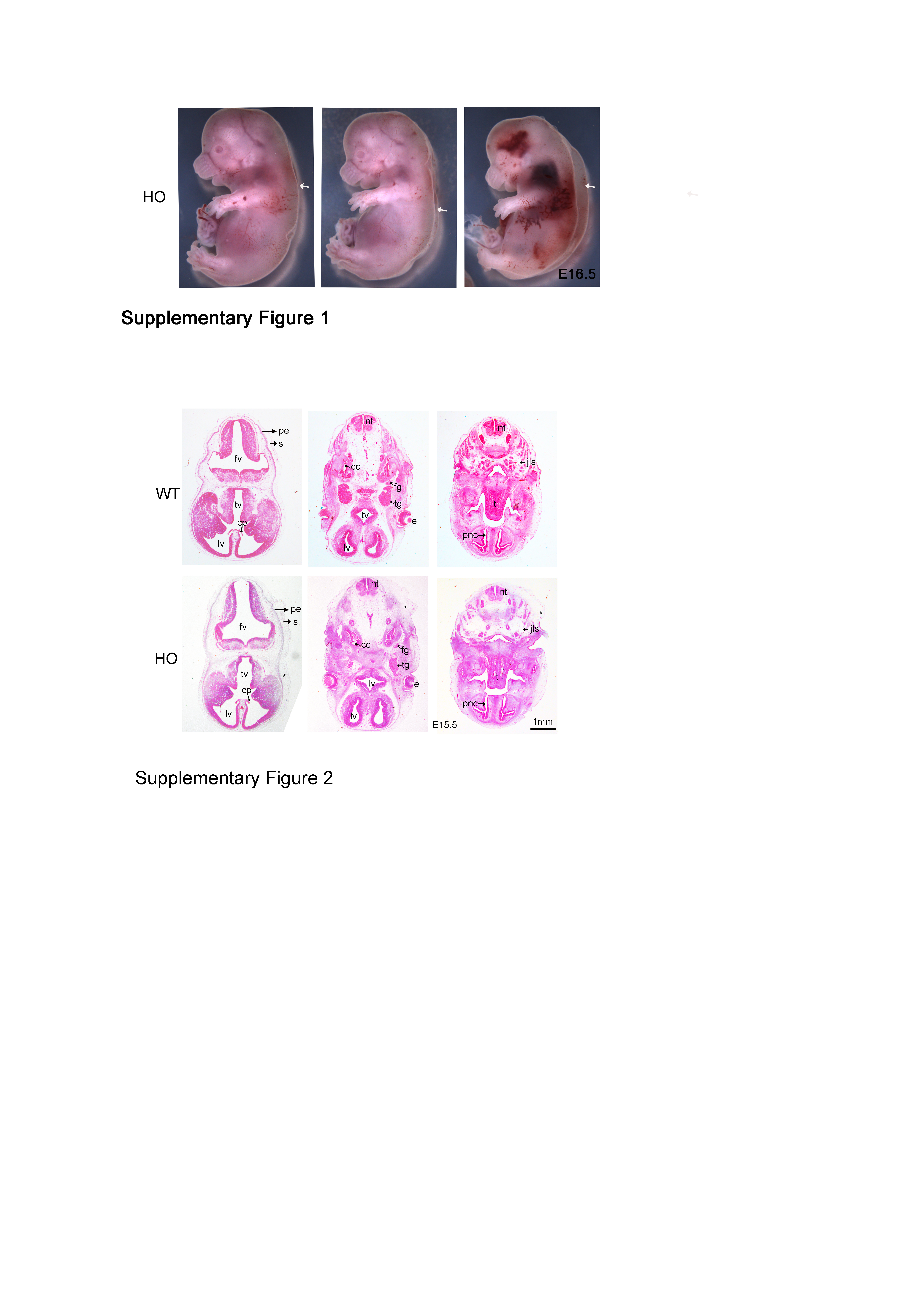

Supplement: Supplementary file 1 [file Data_Sheet_1.zip › Supplementary Figure 1 and 2.jpg]
